# Bioinspired magnetic nanoparticles as multimodal photoacoustic, photothermal and photomechanical contrast agents

**DOI:** 10.1038/s41598-018-37353-5

**Published:** 2019-01-29

**Authors:** Zeid A. Nima, Fumiya Watanabe, Azemat Jamshidi-Parsian, Mustafa Sarimollaoglu, Dmitry A. Nedosekin, Mikyung Han, J. Alex Watts, Alexandru S. Biris, Vladimir P. Zharov, Ekaterina I. Galanzha

**Affiliations:** 1 Center for Integrative Nanotechnology Sciences, the University of Arkansas at Little Rock, Little Rock, AR 72205 USA; 2 Arkansas Nanomedicine Center & Winthrop P. Rockefeller Cancer Institute, University of Arkansas for Medical Sciences, Little Rock, AR 72205 USA; 3 Laboratory of Lymphatic Research, Diagnosis and Therapy (LDT), University of Arkansas for Medical Sciences, Little Rock, AR 72205 USA

## Abstract

Nanoparticles from magnetotactic bacteria have been used in conventional imaging, drug delivery, and magnetic manipulations. Here, we show that these natural nanoparticles and their bioinspired hybrids with near-infrared gold nanorods and folic acid can serve as molecular high-contrast photoacoustic probes for single-cell diagnostics and as photothermal agents for single-cell therapy using laser-induced vapor nanobubbles and magnetic field as significant signal and therapy amplifiers. These theranostics agents enable the detection and photomechanical killing of triple negative breast cancer cells that are resistant to conventional chemotherapy, with just one or a few low-energy laser pulses. In studies *in vivo*, we discovered that circulating tumor cells labeled with the nanohybrids generate transient ultrasharp photoacoustic resonances directly in the bloodstream as the basis for new super-resolution photoacoustic flow cytometry *in vivo*. These properties make natural and bioinspired magnetic nanoparticles promising biocompatible, multimodal, high-contrast, and clinically relevant cellular probes for many *in vitro* and *in vivo* biomedical applications.

## Introduction

Photoacoustic (PA) methods with fast growing applications in spectroscopy, flow cytometry, microscopy, tomography, and image-guided theranostics offer unique features that other optical modalities cannot, including (1) the ability to assess deep (up to 3–5 cm) tissue structures, (2) insensitivity to light scattering and autofluorescent backgrounds, and (3) safety and clinical applicability requiring only low laser energy that is within laser safety standards^1–8^. *In vivo* PA flow cytometry (PAFC), which is currently being used in melanoma, malaria, and stroke related clinical trials, provides the capability to count and molecularly characterize individual fast-moving objects (cells, clots, and nanoparticles) that disseminate by blood, lymph, and cerebrospinal fluid inside humans and animals^2,3,9–13^. The main advantages of *in vivo* PAFC over conventional *in vitro* flow cytometry^14^ include (1) up to 1,000-fold increased sensitivity due to analysis of almost the entire volume of blood (~5 L in humans) compared with ≤0.1% of the volume of blood *in vitro* (typically 1–10 mL), (2) assessing function of single circulating cells in their natural biological environment, and (3) real-time monitoring of changes in functional activity caused by treatment (potentially avoiding a delay in correcting therapy). Compared to fluorescent flow cytometry *in vivo*^15–17^, PAFC provides (1) noninvasive (i.e., through intact skin) counting of fast-moving cells in relatively deep vessels (~1 cm)^10^, (2) targeted theranostics of diseased circulating cells simultaneously with assessment of therapy efficacy, and (3) clinical relevance due to its biocompatibility and safety. To detect and kill low-absorbing circulating cells *in vivo* (e.g., most circulating tumor cells [CTCs]), we have developed molecular targeting that uses highly absorbing nanoparticles (e.g., gold and magnetic) as PA and photothermal (PT) high contrast agents^3^. Using different nanoparticles conjugated with ligands (e.g., antibodies and folic acid) to specific cellular receptors, multicolor PAFC can identify the molecular profile of cells of interest in blood, lymph, and cerebrospinal fluid^2,3,11,13,18^.

While many nanoparticles have been engineered, their possible toxicity often compromises their clinical benefits and imposes new demands on the biocompatible nanoparticles for clinical translation and *in vivo* use^19–21^. One of the promising potential biocompatible PA contrast agent is natural magnetic nanoparticles (nMNPs) that are genetically produced in specific organelles (magnetosomes) of magnetotactic bacteria (MBs)^22–25^. These bioproduced nMNPs are single-domain monocrystalline ferrimagnets (magnetite [Fe_3_O_4_] or greigite [Fe_3_S_4_]) with a high level of purity and crystallinity and a high magnetic moment. They have uniform morphology that is biogenetically controllable and reproducible. In contrast to engineered nanoparticles, nMNPs have a natural phospholipid membrane coating, resulting in good biocompatibility, easy functionalization (because of many amino groups on the surface), a negatively charged surface, and good dispersion in water and saline solutions^26,27^. These unique characteristics are difficult or impossible to achieve in chemically synthetized (i.e., engineered) nanoparticles. As a result, MBs and nMNPs are receiving growing interest in biomedical research. They have already shown superiority over engineered nanoparticles as (1) contrast agents in magnetic-resonance imaging (MRI); (2) therapeutic agents for magnetic hyperthermia of primary tumors, showing larger magnetic losses when converted into heat; and (3) advanced drug carriers^28–32^.

**Figure 1:**
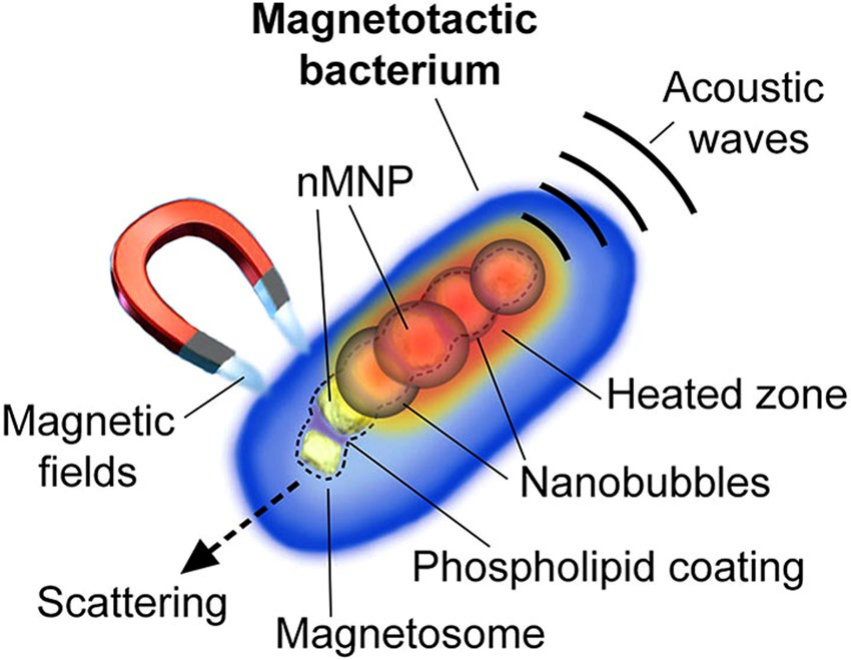
Schematic of a magnetotactic bacterium (MB) as a multimodal contrast agent for PA and PT detection, dark-field imaging, and magnetic manipulations.

However, despite their favorable profile, nMNPs and MBs have never been used in photoacoustics. In this work, we demonstrated that MBs, nMNPs, and their bioinspired hybrids with gold nanorods (GNRs) can be used as advanced high contrast and specific agents in PA and PT spectroscopy, cytometry, and flow cytometry *in vitro* and *in vivo* for the detection, magnetic manipulation, and therapy of single cells (Fig. 1).

## Results

### Magnetotactic bacteria as PA high contrast agents *in vitro*

**Figure 2:**
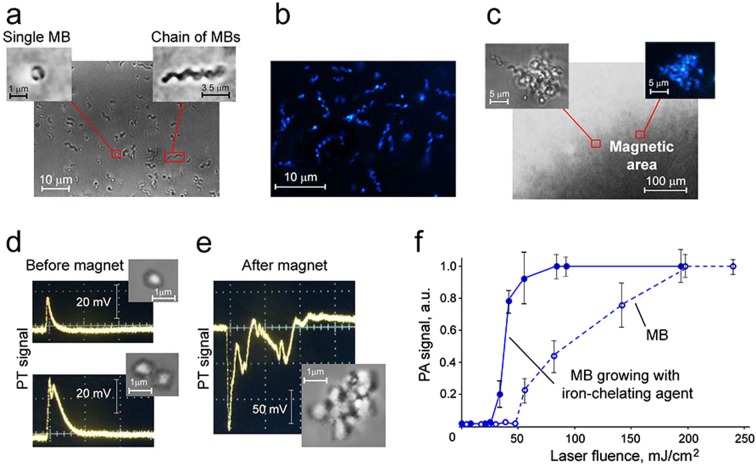
PA and PT detection, imaging, and magnetic manipulations of MBs. (**a**) High-resolution optical images (100×, oil immersion); inserts: round-shaped single MB (left) and chain of MBs (right). (**b**) Dark-field image (60×, oil immersion). (**c**) Image of magnetically trapped MBs (10×); inserts: high resolution optical (left) and dark-field (right) images (both 100×, oil immersion) of magnetically activated clusters of MBs. (**d**) Linear (top) and nonlinear (bottom) PT signal at 670 nm from 1 and 2 MBs. (**e**) Dramatically increased nonlinear PT signals from the magnetically-induced MB cluster. (**f**) PA signal amplitude as a function of laser fluence at 532 nm from MBs growing without (dashed line, open circles) and with (solid line, filled circles) iron-chelating agent. The standard error of the mean for the PA signals is in the range of 7–15%.

High-resolution dark-field microscopy, in combination with optical transmission imaging, demonstrated that MBs with strong light scattering appear as single or a chain of round, small cells (Fig. 2a,b). The tracking of single MBs and their chains in suspension using video monitoring showed chaotic Brownian movements typical for any bacterial cells. However, attachment of the magnet revealed the unique attribute of MBs: directed motion to the magnet area that led to accumulation (trapping) of MBs above the magnet (Fig. 2c). The magnetic forces also stimulated formation of large MB clusters (Fig. 2c, inserts).

To explore the PT and PA properties of MBs *in vitro*, the bacterial suspension was diluted to provide single bacteria (~1 µm in diameter) in the laser beam with a diameter of ~10 µm. In PT detection mode, MBs provided well-detectable PT signals at laser wavelengths of 532 and 670 nm. Each PT signal from individual bacteria was a result of averaging thermal effects from intracellular nMNPs, which are naturally packed into magnetosomes (chain or cluster of nMNPs) inside MBs (Fig. 1). Since clusters of engineered nanoparticles usually provide nonlinear signals with typical negative peaks associated with refraction and scattering of the probe beam on the laser-induced vapor nanobubbles around overheated nanoparticle clusters^33^, we predicted that MBs with natural clusters would also exhibit nonlinear signals. Indeed, our experimental measurements showed that only a few single bacteria had linear PT signals (Fig. 2d, top), with a fast-rising ns-scale peak and slow µs-scale decay; the most PT signals from MBs were nonlinear (Fig. 2d, bottom). Trapping of MBs by the magnet (Fig. 2c) resulted in an increase (up to 10 times) in PT signal amplitude. All PT signals from magnetically trapped MBs were non-linear (Fig. 2e) due to enhancement of local absorption from magnetically induced MB clusters (Fig. 2c, inserts).

Next, we tested PA detection mode. The basic physical processes of PT and PA methods are very similar, but PA has the advanced ability to detect individual cells labeled with nanoparticles *in vivo* in deep tissue^2,11^. As the first step, optimizing the PA parameters of new contrast agents *in vitro* is crucial for future *in vivo* applications. In our study, *in vitro* PA responses from single MBs were measured at different levels of laser energy, from 3 to 1,000 mJ/cm^2^. This allowed us to estimate the threshold of laser fluence for PA detection of single bacteria, which was around 35 mJ/cm^2^. By gradually raising the laser fluence from 80 to 200 mJ/cm^2^, we obtained nonlinear amplification of PA responses, which became saturated after 200 mJ/cm^2^ (Fig. 2f). To increase the PA contrast of MBs, we added an iron-chelating agent (hemoglobin) to the culturing media to enhance production of magnetosomes^34^. As a result, the MBs demonstrated the first PA responses and saturation at 2–2.5 times lower laser fluence (~20–25 mJ/cm^2^ vs. 80–85 mJ/cm^2^) than MBs grown without this agent (Fig. 2f).

### PA characteristics and ultrasharp nonlinear PA resonances from nMNPs and bioinspired hybrids of nMNPs and GNRs (nMNP-GNRs) *in vitro*

**Figure 3:**
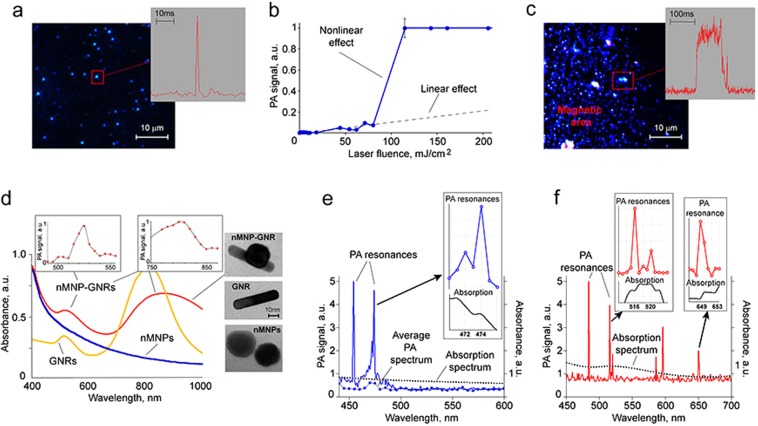
PA characterization of natural (nMNPs) and bioinspired (nMNP-GNRs) nanoparticles. (**a**) Dark-field imaging of nMNPs and typical PA signal from single nMNP (insert) at laser fluence of 115 mJ/cm^2^ and wavelength of 532 nm. (**b**) Linear (dashed line) and nonlinear (solid line) behavior of PA signal amplitudes from nMNPs at different laser fluences (532 nm). (**c**) Dark-field image of magnetically trapped clusters of nMNPs; insert: wide high-amplitude PA signal with multiple peaks from the cluster (laser fluence of 62 mJ/cm^2^ and wavelength of 532 nm). (**d**) Absorption spectra (left) and corresponding TEM images (right) of nMNPs, GNRs, and nMNP-GNR hybrids; inserts: PA spectra at visible (left) and NIR (right) regions. (**e**) Ultrasharp nonlinear PA resonances in PA spectroscopy with increment of Δλ = 1 nm, average PA spectrum with increment of Δλ = 5 nm, and absorption spectrum obtained from nMNPs; insert: high-resolution ultrasharp spectral resonances (top) and corresponding changes in absorption spectrum (bottom). (**f**) Ultrasharp nonlinear PA resonances in PA spectroscopy with increment of Δλ = 1 nm and absorption spectrum obtained from nMNP-GNRs;  inserts: high-resolution ultrasharp resonances in visible (left) and far-red regions (right) and corresponding changes in absorption spectrum. The standard error of the mean for PA signals is in the range of 5–12%.

The nMNPs were extracted from MBs based on previously reported protocols^29,34,35^. However, the purification of nMNPs from bacterial debris is a complex procedure and not always efficient. To overcome this challenge, we developed magnetic-activated sorting of nMNPs using commercially available magnetic MS Columns (MACS). This important step allowed us to quickly and simply achieve pure suspension of nMNPs (Fig. 3a).

To test nMNPs’ use as PA contrast agents, we obtained their PA responses at 532 nm. The typical shape (transient sharp peak) of high-amplitude PA signals (scanning PA cytometry mode) from single nMNPs is shown in Fig. 3a (insert). Measuring PA signals from nMNPs as a function of laser fluence energy showed that the threshold of laser fluence for nonlinear PA detection of single nMNPs was around 8 mJ/cm^2^. At lower energy fluence, the PA signal was linear (Fig. 3b). As the laser fluence increased, the PA signal exhibited nonlinear enhancement and, finally, saturation (plateau) associated with laser-induced vapor nanobubbles around overheated nMNPs and their bleaching of the thermal origin, respectively.

Based on our experience with engineered magnetic nanoparticles, we examined magnetic manipulation of nMNPs to enhance PA signals^3,33^. As expected, we observed magnet-induced trapping of nMNPs (Fig. 3c) and their clustering (Fig. 3c, red square) inside the magnetic area. Magnet-activated clustering led to amplification of PA signals and changes in the signals’ shape and width. Single nMNPs typically created single sharp PA peaks (Fig. 3a, insert), while magnetically induced clusters provided wider PA signals with multiple peaks (Fig. 3c, insert).

Then, the nanoparticles were analyzed with PA and conventional spectroscopy to measure PA and absorption spectra (Fig. 3d and Supplementary Fig. 1). Similar to engineered magnetic nanoparticles^3^, the nMNPs demonstrated relatively high absorption in the visible range, with monotonic decreasing toward far-red and near-infrared (NIR) ranges. The low absorption of nMNPs in the NIR range resulted in low PA signals. However, agents with high NIR contrast are highly desired for *in vivo* applications because the NIR range is known as “window” transparency, providing deeper penetration of light in biotissues. To achieve high NIR contrast, we synthetized bioinspired nanoparticles by decorating nMNPs with GNRs, which have strong NIR absorption (Fig. 3d, right). The efficacy of the binding was confirmed by transmission electron microscopy (TEM) (Fig. 3d, right, top).

Both absorption and PA spectra showed that nMNP-GNRs exhibited (1) high absorption in the visible range, with the peak around 530 nm associated with GNR transverse plasmonic resonances; and (2) strong NIR absorption, with the maximum at 700–900 nm associated with GNR longitudinal plasmonic resonances (Fig. 3d). All three types of nanoparticles were compared, and the results showed that the conventional absorption of nMNP-GNRs in the visible range of 500–550 nm was ~1.3–1.6 times stronger than the absorption of nMNPs and GNRs alone. NIR absorption was very low for nMNPs, high for nMNP-GNRs, and maximal for GNRs. The NIR absorption peak of the nMNP-GNRs was characterized by a spectral red shift (~60 nm) and broadening compared to the NIR peak of GNRs. It should also be noted that the NIR absorption of the hybrids was ~5 times higher than the absorption of nMNPs (Fig. 3d). This relatively high absorption of the hybrids, as well as their magnetic properties (e.g., magnetic trapping), suggests their usefulness as advanced multimodal multifunctional contrast agents for (1) PA detection in visible wavelengths, which is suitable for *in vitro* studies; (2) NIR PA diagnosis, which is preferable for *in vivo* applications; and (3) magnetic manipulations (Table 1).

**Table 1:** A comparison of the major advantages of each type of nanoparticle.

	Nanoparticle Type		
Characteristics	nMNPs	nMNP-GNRs	GNRs
PA contrast in visible range	high	highest	high
PA contrast in NIR range	low	high	high
PA ultrasharp resonances	yes in visible range	yes in visible and NIR ranges	yes in visible and NIR ranges
PT cell therapy	not found	yes	yes
Magnetic manipulation	yes	yes	no
Toxicity at the detectable range of concentrations	not found	depends on GNR concentration	depends on GNR concentration

Next, we studied the ability of nMNPs and nMNP-GNR hybrids to produce ultrasharp nonlinear PA resonances. This unique, advanced phenomenon attributed to nanoparticles is based on laser-induced vapor nanobubbles around overheated nanoparticles that lead to nonlinear spectral and spatial narrowing of PA spectra and, thus, imaging of structures down to 1 nm and 50 nm, respectively^36,37^. Ultrasharp spectral resonances are usually generated near the centers of small absorption peaks of nanoparticles leading to the nanobubble-based amplifications of these peaks. Since nMNPs have high absorption in the visible range (Fig. 3d, black curve), the PA resonances were seen around 450–500 nm. In this range, the PA spectra from the nMNP suspension at a relatively low laser energy of ~2 µJ/pulse and increments of Δλ = 1 nm demonstrated narrow (1–3-nm-wide) peaks with dramatic enhancement in PA signal amplitudes (Fig. 3e). As expected, some ultrasharp PA peaks were matched to the slight increase in absorption on the conventional spectrum (Fig. 3e, insert). Notably, when averaging the PA signals (increments up to Δλ = 5–10 nm), ultrasharp resonances disappeared, and PA spectroscopy at the same energy showed non-detectable signals or signals with low amplitude (Fig. 3e, curve with blue circles). The decreasing or even full disappearance of the resonances can be explained by the laser impact on nanoparticle clusters, in which the spectral position of an absorption maximum can be slightly fluctuated depending on individual nanoparticle spatial locations, especially the distances between them. As a result, during averaging, spectrally fluctuated ultrasharp peaks can be broadened and reduced in amplitude.

Testing PA resonances from nMNP-GNRs using PA spectroscopy with increments of Δλ = 1 nm showed their additional capability to produce narrow peaks in the far-red region, associated with changes in the conventional absorption spectrum (Fig. 3f). Furthermore, we found the correlation of the ultrasharp resonances with the laser energy levels (Supplementary Fig. 2). As expected, the resonances were not produced at very low energy (i.e., in linear mode). Additionally, the resonances did not appear at very high laser pulse energies, likely due to burning of nMNPs and nMNP-GNRs^36^.

### *In vitro* PA contrast of mammalian cells nonspecifically loaded with MBs, nMNPs, and nMNP-GNRs

**Figure 4:**
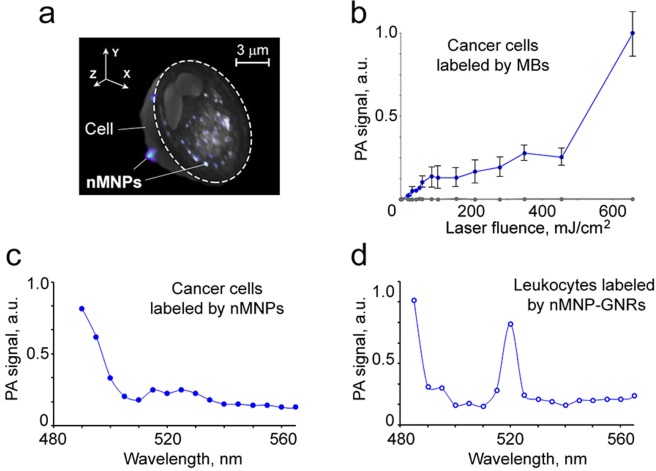
PA assessment of mammalian cells nonspecifically loaded by MB, nMNP, and nMNP-GNR. (**a**) 3D combined dark-field and transmission image of breast cancer cell with cross-sectional display (dashed line). (**b**) PA signal amplitude from breast cancer cells with MBs as a function of laser fluence at 532 nm. (**c**,**d**) PA spectra of breast cancer cells with nMNPs (**c**) and leukocytes with nMNP-GNRs (**d**).

The use of MBs, nMNPs, and nMNP-GNRs as PA contrast agents requires their efficient cellular uptake. For this, leukocytes (normal white blood cells [WBCs]), extracted from mouse blood, and human breast cancer cells (MDA-MB-231 cell line) were incubated with the aforementioned contrast agents and detected using PA cytometry and spectroscopy. Both types of mammalian cells were able to uptake MBs, nMNPs, or nMNP-GNRs. All three contrast agents preferably accumulated in the cytoplasm of cells, as observed via 3D reconstruction of combined optical and dark-field images (Fig. 4a). Only a few small clusters were found on the cell surface.

Scanning PA cytometry demonstrated that most of the labeled cells, in contrast to control (unlabeled) cells, were well-detectable at relatively low laser energy (Fig. 4b–d). For example, the threshold of laser fluence for PA detection of MB-labeled breast cancer cells (Fig. 4b) was around 20 mJ/cm^2^. The average amplitude of PA signals from cells loaded with MBs (0.0121 ± 0.0034 a.u.) was about 10 times higher than the PA signals from unlabeled cells (0.0012 ± 0.0002 a.u.; p < 0.05). As expected, the increasing laser energy caused nonlinear enhancement of PA signals.

PA spectroscopy of the labeled cells showed spectra similar to the PA and absorption spectra of nanoparticles alone (Fig. 4c,d), including higher amplitude signals from cells with nMNP-GNRs at 520 nm (Fig. 4d) compared to cells with nMNPs (Fig. 4c). Notably, PA spectroscopy at increments of Δλ = 1 nm showed ultrasharp resonances at 460–500 nm from cells with nMNPs and random resonances in visible and far-red regions from cells with nMNP-GNR (Supplementary Fig. 3).

Though strong evidence of nMNP biocompatibility^26,27^ and low GNR toxicity has already been reported, additional experiments were done to test mammalian cell viability after incubation with nMNPs as well as with novel nMNP-GNRs at the incubation time and concentrations used in this study (Supplementary Fig. 4). As expected, nMNPs did not change cell viability (94.5 ± 5.7% viable cells) at concentrations up to 24 mg/ml: 10^1^–10^2^ times higher than experimental concentrations (1.5 mg/ml or less). The nMNP-GNR hybrids also demonstrated good biocompatibility at the used concentrations. The viability of nMNP-GNR-treated cells was comparable with the viability of intact cells (no nanoparticles; positive control) and significantly higher than the viability of cells incubated with DMSO (negative control). However, increasing the nMNP-GNR concentration to over 1 mg/ml of GNRs reduced cell viability in a concentration-dependent manner. This reduction is attributable to the GNRs because the same cell behavior was seen during incubation with GNRs alone at a concentration of ≥1 mg/ml per cell, while nMNPs did not change viability even at 24 mg/ml.

### *In vitro* single cancer cell theranostics

**Figure 5:**
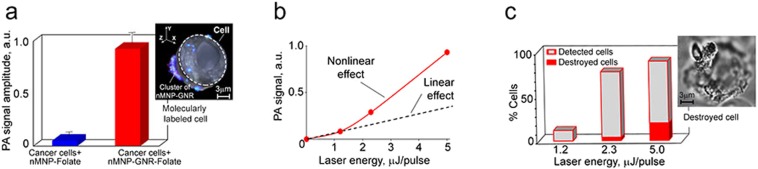
PA molecular detection and targeted therapy of breast cancer cells *in vitro*. (**a**) PA signal from cancer cells labeled by nMNP-folate and nMNP-GNR-folate under a single laser pulse of 5 µJ; insert: 3D image of the breast cancer cell molecularly labeled by nMNP-GNR-folate with cross-sectional display (dashed line). (**b**) PA signal amplitude from cells with nMNP-GNR-folate as a function of laser fluence after one pulse. (**c**) Percentage of detected and destroyed (killed) cancer cells under one laser pulse at different laser fluences; insert shows optical image of destroyed cancer cell after laser therapy. Laser wavelength is 532 nm. Error bars in (**a**) represent the standard error of the mean.

To explore the potential of nMNP-GNRs and nMNPs as molecular contrast agents, they were conjugated with folic acid (nMNP-GNR-folate and nMNP-folate, respectively) and incubated with MDA-MB-231 breast cancer cells, which are triple negative and express folate receptors^38,39^. Labeled cells were analyzed by PA cytometry using single laser pulses with the laser energy from 1 to 5 µJ (Fig. 5a,b). At 5µJ, 80–85% of cells produced high amplitude PA signals associated with their molecular labeling. In comparison, only 3–5% of cells incubated with folate-free nMNPs were detectable using a 5-µJ laser pulse. This is in good agreement with previous reports related to labeling breast cancer cells with folate-conjugated engineered nanoparticles^3,13,33^. PA results were confirmed by dark-field imaging of single cells. The 3D reconstructed images showed that most nMNPs and nMNP-GNRs accumulated on the extracellular membrane as large clusters, likely due to nanoparticle aggregation around cell receptors (Fig. 5a, insert). Some nanoparticles and their small clusters were internalized by cells and localized in cytoplasm. To compare PA contrasts of nMNP-GNR-folate and nMNP-folate, both were incubated with cancer cells under the same parameters and assessed by PA cytometry at 532 nm. The cells with nMNP-GNR-folate produced ~10 times higher PA signals than cells with nMNP-folate (Fig. 5a), clearly demonstrating the superiority of nMNP-GNRs as PA contrast agents.

When the laser pulse fluence was increased, the cells labeled with nMNP-GNR-folate demonstrated typical transformation of linear PA signals into non-linear PA signals (Fig. 5b). In addition, as the laser energy increased, more cells produced PA signals. To minimize the required laser energy level, as is desirable for use in living systems, we applied magnetic forces to induce the clustering of nMNP-GNRs inside cells, leading to PA signal amplification. Thus, only few molecularly attached nMNP-GNRs gained high PA contrast. As a result, undetectable cancer cells with low receptor expression were well-detectable: the number of cells responding to one laser pulse of 2.0–2.5 µJ increased from 75% to 98% after magnet attachment.

Next, we explored the theranostics capability of the nMNPs and nMNP-GNRs. As mentioned in previous reports^2,11^, laser irradiation of cells labeled with engineering nanoparticles can create nanobubbles that produce a highly localized (within 0.5–1 µm) “explosion,” resulting in the mechanical destruction (photomechanical effect) and death of single irradiated cells. This mechanism is very attractive for therapy because it allows targeted killing of circulating diseased cells without damage to the surrounding normal cells^2,11^. To model single CTC theranostics *in vitro*, breast cancer cells were molecularly labeled with nMNP-folate and nMNP-GNR-folate and irradiated by a pulsed laser at increasing laser fluences. Each cell was irradiated by one laser pulse, assuming that only one pulse can be applied to a fast-moving CTC in blood circulation *in vivo*. We found that a single laser pulse (1–5 µJ) was able to kill cancer cells labeled with nMNP-GNR-folate, but this laser energy was not enough to kill cells labeled with nMNP-folate (Fig. 5c). As expected, higher pulse laser energy increased cell killing efficacy. Cell destruction was verified by high-resolution (100x and 60x; oil immersion) optical imaging (Fig. 5c, insert).

These successful experiments *in vitro* prompted us to explore the potential of the novel bioinspired PA contrast agents *in vivo*.

### *In vivo* super-resolution two-color PAFC with transient ultrasharp resonances from CTCs molecularly labeled with nMNP-GNRs

**Figure 6:**
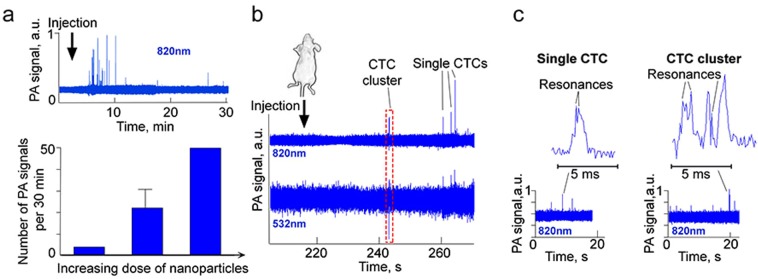
*In vivo* two-color super-resolution PAFC of CTCs labeled with nMNP-GNR-folate in mice. (**a**) Original PA trace (top) and number of PA signals (bottom) after injection of 3 doses of nMNP-GNR-folate: 1^st^: 30 µg Au + 450 µg Fe; 2^nd^: 3 µg Au + 45 µg Fe; 3^rd^: 0.5 µg Au + 4.5 µg Fe. (**b**) PA traces at 820 and 532 nm recorded in a 50-µm artery after injection of labeled cells. (**c**) PA signal with ultrasharp resonances from fast-moving CTC (left) and PA signal with resonances from fast-moving CTC cluster (right); laser wavelength is 820 nm.

*In vivo* assessment of nMNP-GNR-folate as a high-contrast NIR PA molecular agent for CTCs was performed in mouse models using two-color PAFC with visible and NIR lasers with wavelengths of 532 and 820 nm, respectively (Fig. 6).

Real-time PAFC monitoring of a mouse blood vessel showed that injection of nMNP-GNRs produced high-amplitude NIR PA signals, with the count gradually decreasing in time and positively correlating with the number of introduced nanoparticles (Fig. 6a).

To model CTCs *in vivo*, cancer cells were prelabeled with nMNP-GNR-folate and injected in mouse circulation. The investigated ear vessel was monitored noninvasively before (recording of PA background) and after injection. Within a few minutes after injection, transient PA signals associated with single CTCs appeared in the PA traces. The amplitude of these signals at both wavelengths was significantly higher (i.e., positive PA contrast) than the background PA signals from blood before injection (Fig. 6b). The ratio of PA signals from nanoparticle-labeled CTCs to PA background signals from blood was remarkably higher at 820 nm (Fig. 6b, top trace) than at 532 nm (Fig. 6b, bottom trace) because of relatively high blood absorption at 532 nm. These results are in line with our previous data^2,3,10^. Simultaneously, relatively high blood absorption at 532 nm was the basis for detection of the negative transient signals (i.e., below blood background) associated with low-absorbing circulating clots, cell aggregates, clusters, thrombi, or emboli (Fig. 6b, red dashed square). Thus, detection of labeled CTCs was optimal at 820 nm, while identification of clusters of cancer and/or any other cells was optimal at 532 nm.

Similarly to the magnetic manipulation of CTCs with engineered magnetic nanoparticles^3^, the attachment of a magnet over the investigated vessel trapped labeled cells, as evidenced by a PA trace of a “bunch” of high-amplitude signals (Supplementary Fig. 5). Because the PA signals from nMNP-GNRs were significantly enhanced *in vitro* through production of ultrasharp resonances, we hypothesized that this phenomenon occurs *in vivo* as well. To experimentally confirm this, we measured the width of each PA signal and estimated its shape (Fig. 6c). Because a single CTC with many closely located nMNP-GNRs passes a narrow linear laser beam (width of ~3 µm) at a velocity of ~5 mm/s in 40–50-µm mouse ear arteries, the expected PA signal widths from this CTC would be in the range of 0.6–3 ms. However, we observed multiple much narrower peaks, with widths of 50–200 µs, due to nonlinear signal amplification near the center of the laser beam with Gaussian spatial profiles. These peak durations correspond to spatial distances of 100–400 nm (Fig. 6c, top). Considering that typical resolution in linear mode related to the width of a laser beam is around 3 µm, our finding of peaks with widths of 50–200-µs demonstrates super-resolution beyond this limit (6–10 times).

Video monitoring showed that the diameter and character of blood flow in the explored vessels during PA monitoring and magnetic manipulation did not visually change; there were no signs of increasing vascular permeability (e.g., hemorrhages along the vessels).

## Discussion

In this study, we demonstrated for the first time, to our knowledge, the high PA contrast of MBs, nMNPs produced by these bacteria, and bioinspired nMNP-GNR hybrids as a result of advanced decoration of nMNPs by NIR-absorbing GNRs. The major characteristics of nMNPs, GNRs, and nMNP-GNRs that are important for their biomedical applications *in vitro* and *in vivo* are summarized in Table 1.

In order to demonstrate the potential of MBs and nMNPs as multimodal and multifunctional PA and PT contrast agents, we used advanced *in vitro* PA and PT cytometry, *in vitro* PA spectroscopy, and *in vivo* PAFC together with magnetic trapping, absorption spectroscopy, TEM, and high-resolution dark-field microscopy. The analysis of the obtained results allowed us to conclude that the high PA contrast of MBs is associated with the nanobubble-based enhancement of nonlinear PA signals around nMNP clusters^2,11^. These clusters are naturally present inside MBs, which contain magnetosomes that are basically clusters of nMNPs^22–25^. High PA contrast could make PA methods useful for assessing the efficacy of drug delivery into diseased tissue (e.g., primary tumor) by MBs^32^.

Similar signal amplification was obtained in PT mode. These nonlinear phenomena, along with the high sensitivity of PT cytometry, provided reliable detection of MBs not only in the visible range at 532 nm, where all magnetic nanomaterials have relatively high absorption, but also in the far-red region at 670 nm.

Purification and testing of genetically produced nMNPs from MBs allowed us to experimentally demonstrate that nMNP behavior is similar to chemically engineered magnetic nanoparticles^3^. Specifically, linear PA signals from a solution of single nMNPs at low laser energy became non-linear amplified signals as laser energy increased. The benefit of nMNPs over engineered magnetic nanoparticles is that the threshold of laser fluence for PA detection of nMNPs was approximately 2 times lower than the threshold for engineered magnetic nanoparticles (20 mJ/cm^2^ for nMNPs vs. 50 mJ/cm^2^ for engineered magnetic nanoparticles^3^ at the same concentration). In addition, nMNPs have well-documented biocompatibility^26,27^, which was confirmed in this study.

However, despite many advantages, NIR absorption and, therefore, PA contrast of MBs and nMNPs in the “window of transparency” desirable for *in vivo* applications are weak. We overcame this challenge by synthesizing bioinspired nMNPs with NIR-absorbing GNRs. These bioinspired nanostructures have high NIR absorption and plasmonic effects^3,36,37^ that maximize PA signals and, as a result, have potential for PA detection and photomechanical killing of cells at the relatively low laser energy that is safe for biotissues. Our experimental results confirmed that the nMNP-GNR hybrids had high PA contrast in both visible (around 520 nm) and NIR (around 800–850 nm) wavelengths—quite enough for both molecular diagnosis and targeted therapy of single cancer cells. In our studies, a single laser pulse was successfully used to detect almost all breast cancer cells labeled with folate-conjugated nMNP-GNRs and to destroy some of them, thus achieving single cell theranostics. A comparison between cancer cells molecularly labeled with nMNPs and with nMNP-GNRs clearly demonstrated the advantage of nMNP-GNRs as PA contrast agents for *in vivo* use. A cell viability assay showed no detectable toxicity effects for nMNPs and low toxicity for nMNP-GNRs, which is in line with previous reports^26,27^. Though the bioinspired nMNP-GNR hybrids showed some toxicity at high concentrations compared to nMNPs, the superiorly attractive characteristics of the hybrids (Table 1) and the high PA contrast of nMNP-GNRs at non-toxic concentrations make them the advanced contrast agents for *in vivo* applications.

Furthermore, it was shown that PA contrasts of MBs, nMNPs, and nMNP-GNRs can be enhanced by three approaches. (First) Magnetically activated trapping of these contrast agents facilitated an additional increase of PA and PT contrasts by causing nanoparticle clustering that eventually led to an increased number of PA-detectable cells labeled with nMNPs and nMNP-GNRs. (Second) Significant enhancement of MB PA contrast was achieved by simply adding iron-chelating agents to the growth media. (Third) Our experiments showed that clustering was enhanced if nanoparticles were conjugated with cell-specific markers (e.g., folate for breast cancer cells) and molecularly attached to the cellular receptors. It should be noted that cluster formations have been reported for cells molecularly labeled by various engineered nanoparticles, and for melanoma cells with natural melanin^10,33^. Clearly clustering was expected for MBs and nMNPs but it was not experimentally evidenced. Our study experimentally measured the magnetically activated enhancement of PA contrast from nMNP clusters for the first time.

In order to define the applicability of nMNP-GNRs for noninvasive single circulating cell analysis *in vivo*, we injected these nanoparticles in a mouse circulation and observed high-amplitude PA signals above blood background. The number of signals was concentration and time dependent. We successfully demonstrated detection of single CTCs—and, likely, their aggregates—molecularly labeled with nMNP-GNR-folate using two-color PAFC (532 nm and 820 nm) with positive and negative contrasts. The NIR laser was preferable for detection of CTCs with nMNP-GNR-folate because the PA blood background was minimal. Simultaneously, the 532-nm visible laser provided relatively high PA background from blood due to high absorption of hemoglobin; therefore, it was ideal for identifying low-absorbing clots (cell aggregates, clusters, thrombi) by negative contrast (i.e., the contrast below background of blood), which aligns with our previous reports^2,11^.

In our previous studies with engineered gold-based nanoparticles *in vitro*, we observed dramatically increased sensitivity and detection specificity of nanoparticle-labeled cells by monitoring PA nonlinear responses and ultrasharp resonances^36^. In the current study we, for the first time, showed spectral ultrasharp high-amplitude PA signals from MBs, nMNPs, and nMNP-GNRs. We discovered PA ultrasharp resonances *in vivo*. Specifically, we found that CTCs labeled with the nMNP-GNR-folate produced transient PA signals with narrow, high-amplitude nonlinear resonances when passed over by the PAFC laser beam. The origin of these signals is associated with spatial ultrasharp resonances generated by nanoparticles under laser exposure^36^. To our knowledge, this is the first demonstration of spatial ultrasharp resonances from circulating objects *in vivo*.

Since ultrasharp resonances significantly amplify PA resolution, sensitivity, and specificity, their discovery in flow *in vivo* should permit super-resolution PAFC. In biomedical research, super-resolution PAFC can improve identification of single cells based on amplification of PA signals. In this study, the laser fluence levels of PAFC, which were used to catch high-amplitude signals from CTCs above the blood background, were safe for the surrounding healthy tissue. No marked changes in mouse behavior and blood vessel function were revealed under laser exposure.

In conclusion, our results demonstrated that MBs, nMNPs, and nMNP-GNRs are new, advanced high PA contrast agents with applications in scanning and flow cytometry as well as in PA and PT imaging. The natural origin of these low toxicity, biocompatible contrast agents^26,27^, along with the safety of PAFC as a diagnostic platform^11^, provides great benefits and high potential for clinical translation toward theranostics of CTCs and other circulating and static diseased cells *in vivo*. Furthermore, PA signals from nMNPs and nMNP-GNRs can be dramatically enhanced by ultrasharp resonances both *in vitro* and *in vivo*. These findings are crucial for improvement of single static and circulating cell analysis *in vitro* and *in vivo*.

A key advantage of *in vivo* super-resolution PAFC with nMNP-GNRs as contrast agents is the integrative multi-parameter and multi-functional assessment of circulating objects using safe laser and nanoparticle parameters. Additional advantages include, but are not limited to, (1) real-time count of labeled cells; (2) defining their molecular profile; (3) identifying cell aggregation (aggregates of CTCs and/or WBCs and/or platelets) that often facilitate the progression of diseases (e.g., cancer and cardiovascular disorders); (4) the magnetic trapping of labeled cells (e.g., CTCs); and (5) killing of diseased circulating cells.

Future systematic studies will be undertaken to define optimal PA parameters for diagnosis and therapy of CTCs in preclinical animal models *in vivo* with inoculated and spontaneous tumors. As a broad impact, the high PA contrast of MBs and nMNPs, combined with their previous successful use for MRI, drug delivery, and thermal therapy^28–32^ and as a potential genetically encoded probe^40^, makes them attractive contrast agents for various *in vitro* and *in vivo* biomedical applications. Furthermore, introducing MBs and nMNPs as PA contrast agents may increase their multimodality and offer new flexibility for selection of detection methods.

## Methods

### MB strain and culture

*Magnetospirillum magneticum* AMB-1 was purchased from the American Type Culture Collection (ATCC #700264) and grown microaerobically in the Revised Magnetic Spirillum Growth Medium (MSGM; ATCC #1653) in a dark room at 26–27 °C.

160 µL of 100 µM hemoglobin solution was added to 50 mL of the bacterial growth medium to act as an iron-chelating agent^34^. The MBs with and without iron-chelating agents were incubated 7–10 days before analysis. Then, MBs were harvested by centrifugation (4,000 rpm; 40 min) and resuspended in phosphate buffer saline (PBS).

### Isolation and magnet-activated sorting of nMNPs from MBs

To isolate nMNPs, the MBs were disrupted by ultrasonication with the cooler using an ultrasound bath (Model 2200, Branson). Then, magnetic MACS MS Columns (Miltenyi Biotec) were used to remove cellular debris and prepare a pure suspension of nMNPs. The purity of nMNPs was confirmed by TEM imaging. The solution with sorted nMNPs was collected in a tube, washed by centrifuging (8,000 rpm, 15 min), and resuspended in PBS at a concentration up to 15 mg/ml for further analysis and synthesis of bioinspired hybrids. To test toxicity, the maximum concentration of nMNPs was 24 mg/ml.

### Bioconjugation of nMNPs with folate

For bioconjugation, nMNPs were sonicated for 15 minutes using a bath sonicator, then 300 μl of nanoparticles were added to 200 μl of (25 mg/ml) folate solution. The reactants were left on shaking for 2 hours. Upon completion of the reaction, the solution was centrifuged at 10,000 rpm for 20 minutes. Then, the precipitate (nMNP-folate) was washed with distillate water several times and resuspended in PBS.

### Synthesis of nMNP-GNR hybrids

Conventional GNRs with a maximal absorption at 820 nm were purchased from Nanopartz Inc. (Loveland, CO). The GNRs and nMNPs were sonicated for 15 minutes using a bath sonicator. Next, 4-mercaptobenzoic acid was prepared with a 10-mM stock solution; in a separate conical flask containing 5 ml of GNRs, 5 µl of 4-mercaptobenzoic acid was added and stirred for 3 hours at 45 °C. The solution was centrifuged for 30 minutes at 10,000 rpm to remove excess chemicals. After that, a two-step conjugation assay was performed to bind the carboxylated GNRs with nMNPs; 100 μl of purified carboxylated nanorods (GNR\4-mercaptobenzoic acid) were conjugated with 300 μl of nMNPs and 200 μl of (25 mg/mL) folate to obtain GNR\4-mercaptobenzoic acid\nMNP\folate. The reaction took place within 4 hours of shaking at room temperature. Upon completion, the solution was centrifuged at 10,000 rpm for 20 minutes. The precipitate (nMNP-GNR-folate) was then washed with distilled water several times and resuspended in PBS.

### Multifunctional integrated PA and PT technical platform

The basic principle and details of our technical platform integrating scanning cytometry *in vitro*, PA spectroscopy, PT cytometry, magnetic and optical imaging modules, and *in vivo* PAFC and imaging have been described in our previous reports^2,3,9–13,33,36,41^ and the Supplementary Note. To image MBs, nMNPs, nMNP-GNRs, and mammalian cells *in vitro* and in blood vessels *in vivo*, the aforementioned setup was integrated with high-resolution optical transmission and dark-field modules (see details in Supplementary Note). For 3D reconstruction, dark-field and optical images were taken together over a 20-μm depth in 1.0-μm steps, giving up to 20 scans of the same cell. To magnetically manipulate the MBs, nMNPs, and nMNP-GNRs, the local permanent magnetic field was provided by a cylindrical neodymium-iron-boron (NdFeB) magnet with Ni-Cu-Ni coating, a diameter of 3.2 mm, length of 9.5 mm, and surface field strength of 0.39 Tesla (MAGCRAFT, Vienna, VA)^3^. The magnet was attached to the bottom of the slide for 30 minutes or to the skin of the mouse ear, close to the detection area, as we previously reported^3^.

### Absorption spectroscopy

Following our previously reported procedures^13^, the optical absorption spectra of the nMNPs, GNRs, nMNP-GNRs and cells were acquired by an Ultrospec 3300 PRO (Amersham Biosciences, Ltd., UK) spectrophotometer in a 1-cm cuvette, operating in the wavelength range 200–1100 nm. The measurements were conducted in increments of Δλ = 1 nm. The absorption of each sample was normalized to the absorption of PBS and water.

### Transmission electron microscopy (TEM) analysis

A few drops of the solutions containing the samples of nMNPs or nMNP-GNRs were deposited on holey carbon-coated copper TEM grids, which were then sufficiently dried before being inserted into a JEM 2100F TEM (JEOL USA, Peabody, MS, USA) equipped with a field emission gun. TEM imaging was performed at 80 kV, and all images were collected in bright-field mode using a Gatan (Pleasanton, CA, USA) US1000 CCD camera.

### Mammalian cells and their incubation with nMNPs and nMNP-GNRs

Human breast cancer cells (MDA-MB-231 cell line, ATCC) were cultured as per the vendor’s specifications. For measurements, the viable cells were resuspended in PBS. WBCs were prepared from blood samples of donor mice using the standard protocol through centrifugation (3,000 rpm for 15 min). The layer of WBCs was collected in a separate tube. The residual erythrocytes were removed with Lysis Buffer (eBioscience*™)* using the vendor’s protocol. The WBCs were washed via centrifugation at 1,000 × g for 10 minutes at room temperature. The pellet was then resuspended in PBS for further analysis.

The cell suspensions were incubated with MBs, nMNPs, nMNP-folate, nMNP-GNRs, and nMNP-GNR-folate at 37 °C for 45 minutes (shaking) then centrifuged (1,400 rpm; 5 min) to wash out unbound nanoparticles. In *in vitro* studies, equal aliquots (~8 µL) of the cell suspension at a concentration of ~10^6^ cells per 1 mL were placed in individual wells (S-24737, Molecular Probes) attached to the microscope slide and analyzed by methods that included PA and PT cytometry, and dark-field and conventional transmission (bright field) optical imaging. The dark-field and optical images of labeled cells were taken either for alive cells (immediately after labeling) or fixed cells. For fixation, cells in suspension were incubated with 4% formaldehyde solution (1:1) for 1 hour at room temperature, washed by centrifugation (1,400 rpm; 5 min), and resuspended in PBS.

### Cell viability

The percentage of viable cancer cells was assessed by a Trypan Blue test after incubation with nMNPs, GNRs, and nMNP-GNRs at different concentrations (e.g., 1.0–1.0 × 10^−3^ mg/ml of Au and 15–1.5 × 10^−2^ Fe for nMNP-GNRs; 1.0–1.0 × 10^−3^ mg/ml of Au for GNRs and 24.0–1.5 × 10^−2^ Fe for nMNPs) for 45 minutes. The results were compared with the viability of untreated cells (PBS) under the same conditions (positive control group). Cells incubated with the DMCO solution were used as the negative control.

### Mouse model

*In vivo* experiments were done with mice in accordance with protocols approved by the UAMS Institutional Animal Care and Use Committee. Mice (nu/nu mice weighing 20–25 g) were purchased from a commercial source. The mice were anesthetized with isoflurane and placed on a heated microscope stage (38 °C). To explore PA detection *in vivo*, three doses of nMNP-GNRs (1^st^: 30 µg Au + 450 µg Fe, 2^nd^: 3 µg Au + 45 µg Fe, and 3^rd^: 0.5 µg Au + 4.5 µg Fe) in 30 µl of PBS were introduced in circulation of nine mice (3 mice per dose). To detect CTCs, breast cancer cells prelabeled with nMNP-GNR-folate were injected into mouse circulation (10^5^ cells in 30 µl of PBS per injection). For PAFC, the laser beam was positioned over the projection of an ear blood vessel (artery with diameter of ~50 µm) while the ultrasound transducer was gently attached to skin close to the point of detection. The area where the transducer was placed was moistened by warm water (or acoustic gel) to facilitate transferring acoustic signals. Videomicroscopy was used to ensure that the laser and/or nanoparticles did not affect vascular function.

### Statistical analysis

Data for each endpoint was summarized by group as means and standard errors, then compared. The differences were considered statistically significant if *P* ≤ 0.05. To provide sufficient material for statistical analysis, up to 50 measurements were performed for each *in vitro* experiment; 3-4 animals were used for *in vivo* monitoring. To ensure robust and unbiased results, *in vitro* measurements were triplicated.

## Supplementary information

Supplementary Information

## Data Availability

The authors declare that relevant data supporting the findings of this study are available on request.
