# Supplementary material for: Bioinspired magnetic nanoparticles as multimodal photoacoustic, photothermal and photomechanical contrast agents
Source: Sci Rep. 2019 Jan 29;9:887. doi: 10.1038/s41598-018-37353-5 (PMC6351522; doi:10.1038/s41598-018-37353-5)
Supplement: Supplementary file 1 — Supplementary Information [file 41598_2018_37353_MOESM1_ESM.docx]

Supplementary information

**Bioinspired magnetic nanoparticles as multimodal photoacoustic, photothermal and photomechanical contrast agents**

Zeid A. Nima, Fumiya Watanabe, Azemat Jamshidi-Parsian, Mustafa Sarimollaoglu, Dmitry A. Nedosekin, Mikyung Han, J. Alex Watts, Alexandru S. Biris, Vladimir P. Zharov,

Ekaterina I. Galanzha

**Supplementary Figures**


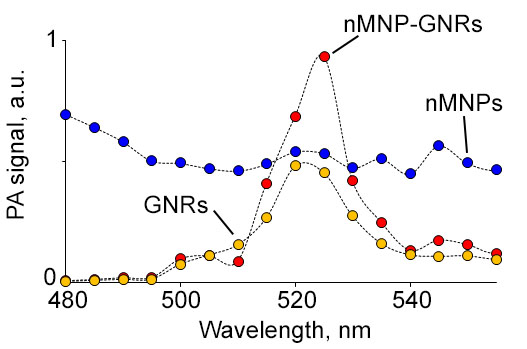


**Supplementary Figure 1**. PA spectra of nMNPs, GNRs, and their hybrids.


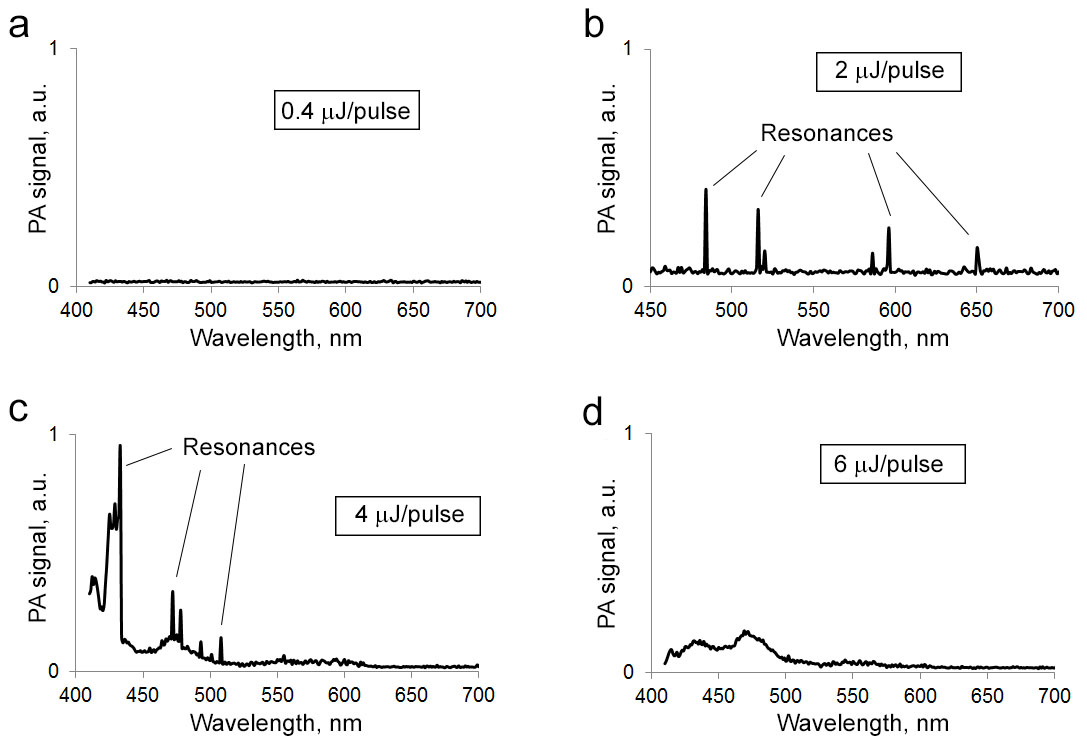


**Supplementary Figure 2**. Ultrasharp nonlinear PA resonances in PA spectroscopy with increment of Δλ = 1 nm obtained from nMNP-GNRs at different laser energies.


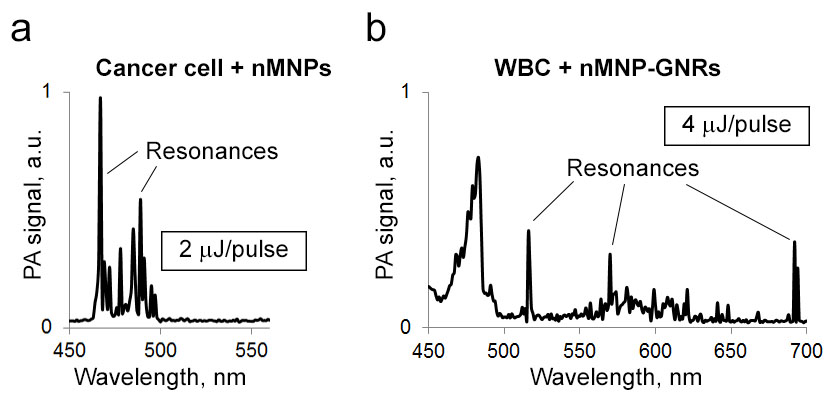


**Supplementary Figure 3**. Ultrasharp nonlinear PA resonances from mammalian cells labeled with natural and bioinspired nanoparticles. (**a**) PA spectroscopy with increment of Δλ = 1 nm obtained from the human breast cancer cell with nMNP. (**b**) PA spectroscopy with increment of Δλ = 1 nm obtained from the mouse white blood cell with nMNP-GNRs.


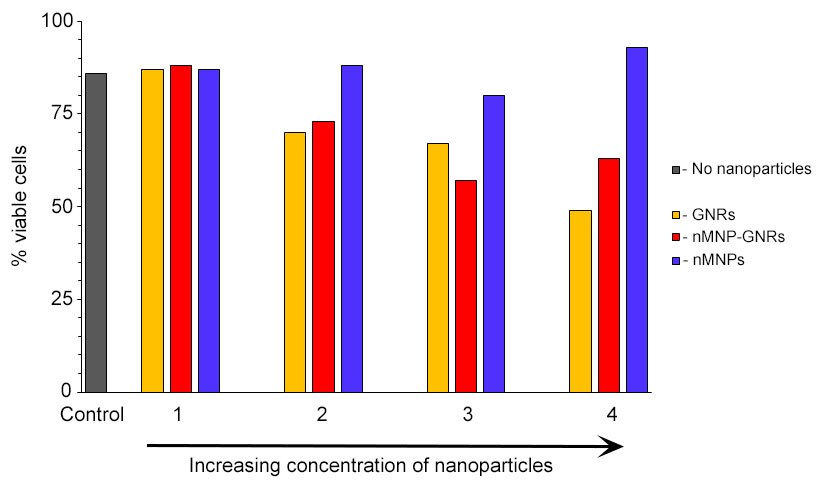


**Supplementary Figure 4**. Viability of breast cancer cells after 45-min incubation with nanoparticle solution containing: (**1**) 1.0×10^-3^ mg/mL Au and 1.5×10^-2^ mg/mL Fe; (**2**) 1.0×10^-2^ mg/mL Au and 1.5×10^-1^ mg/mL Fe; (**3**) 1.0×10^-1^ mg/mL Au and 1.5 mg/mL Fe; and (**4**) 1.0 mg/mL Au and 15.0 mg/mL Fe for GNRs and nMNP-GNRs and 24.0 mg/mL Fe for nMNPs. The viability of cancer cells after 45-min incubation in PBS is shown as positive control.


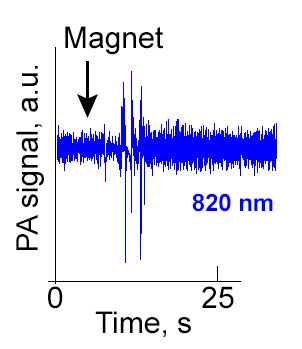


**Supplementary Figure 5**. *In vivo* noninvasive (i.e., through skin) intravascular magnetic trapping of CTCs in mouse circulation detected in PA flow cytometry (PAFC) trace as the “chain” of high-amplitude positive and negative signals.

**Supplementary Note**

**Integrated technical platform**

The basic principle and details of our technical platform integrating scanning cytometry *in vitro*, photoacoustic (PA) spectroscopy, photothermal (PT) cytometry, magnetic and optical imaging modules, and *in vivo* PA flow cytometry (PAFC) and imaging have been described in our previous reports.^2-3,9-13,34,36,41^ Briefly, the scanning PA and PT microscope was built on the platform of an upright Olympus IX81 microscope (Olympus America, Inc., Central Valley, PA) and equipped with high-pulse-repetition-rate nanosecond lasers with the following wavelength, maximal pulse energy, pulse width, and pulse-repetition rate parameters: 1): 532 nm, 30 μJ, 5 ns, and up to 100 kHz, respectively (model: LUCE 532, Bright Solutions, Cura Carpignano, Italy); 2) 671 nm, 35 μJ, 25 ns, and 100 kHz, respectively (model: QL671-500, CrystaLaser, Reno, NV); and 3) 820 nm, 75 μJ, 8 ns, and 30 kHz, respectively (model: LUCE 820, Bright Solutions). A 2.25 MHz ultrasound transducer (V323-SU, Panametrics NDT Inc.) was used to acquire PA signals from the sample in transmission mode (on top of the slide). The amplified (50 kHz–5 MHz bandwidth amplification, 54 dB gain, model 5662, Panametrics NDT Inc.) signal was acquired by a digitizer (PCI-5124, National Instruments, Austin, TX), which also controlled sample scanning by a two-dimensional (2 D; X–Y) translation stage (H117 ProScan II, Prior Scientific, Rockland, MA).^2,11^

For PA spectroscopy, a tunable laser-based optical parametric oscillator (OPO, Opolette HR 355 LD, OPOTEK, Carlsbad, CA) was used with the following parameters: spectral range: 410–2,200 nm; pulse width: 5 ns; pulse repetition rate: 100 Hz; line width: 0.5 nm; pulse energy: up to 2 mJ; fluence range: 1–104 mJ/cm^2^; and pulse energy stability: 3–5%. The energy of each OPO pulse was controlled by an energy meter (PE10-SH, OPHIR, Logan, UT).^2,3,10,36^

In PT thermal lens mode in scanning PT cytometry, pump (OPO) laser-induced temperature-dependent variations of the refractive index caused an unfocused collinear He-Ne laser probe beam (633 nm, 1.4 mW) and, hence, a reduction in the beam’s intensity at its center. This was detected by a photodiode with a pinhole (referred to as PT signals). ^2,3,10,36^

The PAFC for *in vivo* measurements uses a similar platform to PA and PT cytometry. Specifically, PAFC irradiates selected vessels with short laser pulses, then uses an ultrasound transducer attached to the skin to conduct time-resolved detection of laser-induced acoustic waves (PA signals). In our *in vivo* experiment, we used a laser with a pulse-repetition rate of 10 kHz and wavelength of 820 nm, which corresponds to the high absorption of nMNP-GNRs in the NIR range. The laser energy levels were adjusted to provide a readable background from blood compared to the skin and, simultaneously, to prevent photodamage to skin and vessels (controlled by optical videomicroscopy). The laser beam(s) was navigated on a selected blood vessel under optical imaging guidance and focused into a linear configuration (from 4.5 μm × 40 μm to 8 μm × 80 μm) across the vessel (i.e., blood flow direction). The PA signals were detected by ultrasound transducers (an unfocused XMS-310 transducer with a 10-MHz frequency band or a focused V316-SM transducer with a 20-MHz frequency band; both from Panametrics NDT/Olympus, Waltham, MA) and then amplified (model 5662 or 5678: Panametrics NDT). The PA signals were digitized with a high-speed analog-to-digital converter board, and then peak-to-peak amplitudes of the acquired PA signals were presented as PA signal traces, analogous to conventional flow cytometry. Real-time and post-processing operations were performed with MATLAB v. 7.0.1 software (MathWorks, Natick, MA). Signal-to-noise ratio (SNR) was determined by the ratio of flash (transient) PA signals from single strongly absorbing labeled CTCs to the background from blood in the detection volume and to noise of different origins (e.g., electronic, acoustic, fluctuating erythrocyte numbers, instability of laser energy).^2-3,9-13,34^

To image MBs, nMNPs, nMNP-GNRs, and mammalian cells *in vitro* and in blood vessels *in vivo*, the aforementioned setup was integrated with high-resolution optical (transmission) and dark-field modules. The optical module used a cooled, color CCD camera (DP72, Olympus-NDT), a high-speed (up to 40,000 frames per second) CMOS digital camera (MV-D1024-160-CL8: Photonfocus AG, Lachen, Switzerland), and a Cascade 650 CCD camera (Photometrics, Tucson, AZ). ^3,10,12,34^

Dark-field microscopy was performed using the same microscope with an enhanced illuminator (CytoViva Inc., Auburn, AL). The illuminator featured a CytoViva 150 condenser and a fiber optic light guide connected to a Solarc 24-W metal halide light source (Welch Allyn, Skaneateles Falls, NY). The sample image was acquired using a 100× oil objective with an iris (Olympus UPlanAPO fluorite, N.A. 1.35–0.55) by a high-resolution color camera (DP72, Olympus America Inc.). The illuminator dramatically increases the contrast of light-scattered nanoparticles inside cells.^41^
